# Recommendation for Photographic Documentation of Safe Laparoscopic Cholecystectomy

**DOI:** 10.1007/s00268-020-05776-9

**Published:** 2020-09-04

**Authors:** Maciej Sebastian, Agata Sebastian, Jerzy Rudnicki

**Affiliations:** 1 Department of General, Minimally Invasive and Endocrine Surgery, Wroclaw Medical University, Borowska Street 213, Wrocław, Poland; 2 Department of Rheumatology and Internal Medicine, Wroclaw Medical University, Borowska Street 213, Wrocław, Poland

## Abstract

**Background:**

Bile duct injury and vasculobiliary injury are possible complications during laparoscopic cholecystectomy which can lead to increased morbidity, mortality, costs of hospitalization and litigation. Proper documentation of the critical view of safety and safe plane of dissection may play a crucial role for archivization, teaching and medicolegal purposes.

**Methods:**

The study group consisted of 100 patients with symptomatic cholecystolithiasis qualified for laparoscopic cholecystectomy. The critical view of safety was documented on two photographs and safe plain of dissection obtained with laparoscopic ultrasound was documented on one photograph as well as the whole procedure was recorded. The photographs were printed in the operating theatre and videos were stored on an external hard drive.

**Results:**

The mean time to obtain and analyse photographs was significantly shorter than video, and the size of the stored data was significantly smaller for photographs than videos. The cost of one documentation procedure was significantly lower for video than photographs. Critical view of safety was obtained in 91 patients, and laparoscopic ultrasound was successful in 99 patients. The conversion rate was 2%, and fundus-first cholecystectomies were performed in 6% of patients. We did not observe any biliary and vascular complications.

**Conclusions:**

Photographic documentation of the critical view of safety and safe plane of dissection should be an inherent part of laparoscopic cholecystectomy. Our proposal of documentation prepared in the operating theatre and stored in the patient’s documentation is an example of an easy, fast and cheap method of data archivization.

## Introduction

Laparoscopic cholecystectomy (LC) is nowadays the gold standard in the treatment of gallstone disease [1, 2]. The most dreaded complication of cholecystectomy is a bile duct injury (BDI) which rate has improved since introduction of LC from 1–1.5% to 0.08–0.3% reported recently what may be associated with increased experience, number of LCs performed beyond the “learning curve” and better instrumentation [3–7]. The critical view of safety (CVS) is a generally accepted method to identify the cystic duct and cystic artery during LC [8]. Laparoscopic ultrasound (LUS) along with intraoperative cholangiography (IOC) is adjunct method of intraoperative visualization especially in doubtful cases [9]. LUS is non-invasive and non-irradiating, visualize vascular structures and may be repeated as many times as it is needed what makes this method more safe and useful than IOC [10]. The proper documentation of obtained CVS and safe plane of dissection may be crucial for learning and teaching purposes and be the source of valuable evidence in the possible litigation process [11–14]. The possible methods of data storage are classical photography and/or video records [12–14]. A very important issue for a surgeon who performs LC is that a method of documentation should be effective, easy-stored, readable, technically sound, cheap and time-efficient with the possibility to finish the whole process of archivization in the operating theatre.

## Materials and methods

The study group finally consisted of 100 patients (61 women and 39 men) operated on between February 2019 and April 2020 in one Department of Surgery. Inclusion criterium for the study was the symptomatic cholecystolithiasis. Exclusion criterium was the pre- or post-operatively diagnosed cancer of the gallbladder, pre-operative acute cholecystitis and previous operations in the abdominal cavity. The demographic and clinical features of the study group are presented in Table 1. The intraoperative identification of the cystic duct and cystic artery was based on CVS, and safe plane of dissection was defined with LUS. Three and always three criteria were required to achieve the CVS:

**Table 1:** Characteristics of the study group according to the age, operating time, length of hospital stay after surgery, reasons for conversion, number of successful CVS and LUS, fundus-first and subtotal LC and cholecystostomies

Parameter	Age [years]	Operating time [min]	Length of hospital stay after surgery [days]	Reason for conversion				Number of successful CVS	Number of successful LUS	Number of fundus-first LC	Number of subtotal LC	Number of cholecystostomies
				Total	Chronic inflammation and fibrosis	Vascular injury	Bile duct injury					
Number of patients	100			2	2	0	0	91	99	6	1	1
Mean	53	54	2.3									
Standard deviation	12.8	11.8	0.5									
Minimum	26	38	2									
Maximum	84	94	4									
Median	53.5	54	2									

CVS-critical view of safety

LC-laparoscopic cholecystectomy

LUS-laparoscopic ultrasound

I The hepatocystic triangle is cleared of fat and fibrous tissue.
II The lower one-third of the gallbladder is separated from the liver to expose the cystic plate.
III Two and only two structures should be seen entering the gallbladder.

**Fig. 1:**
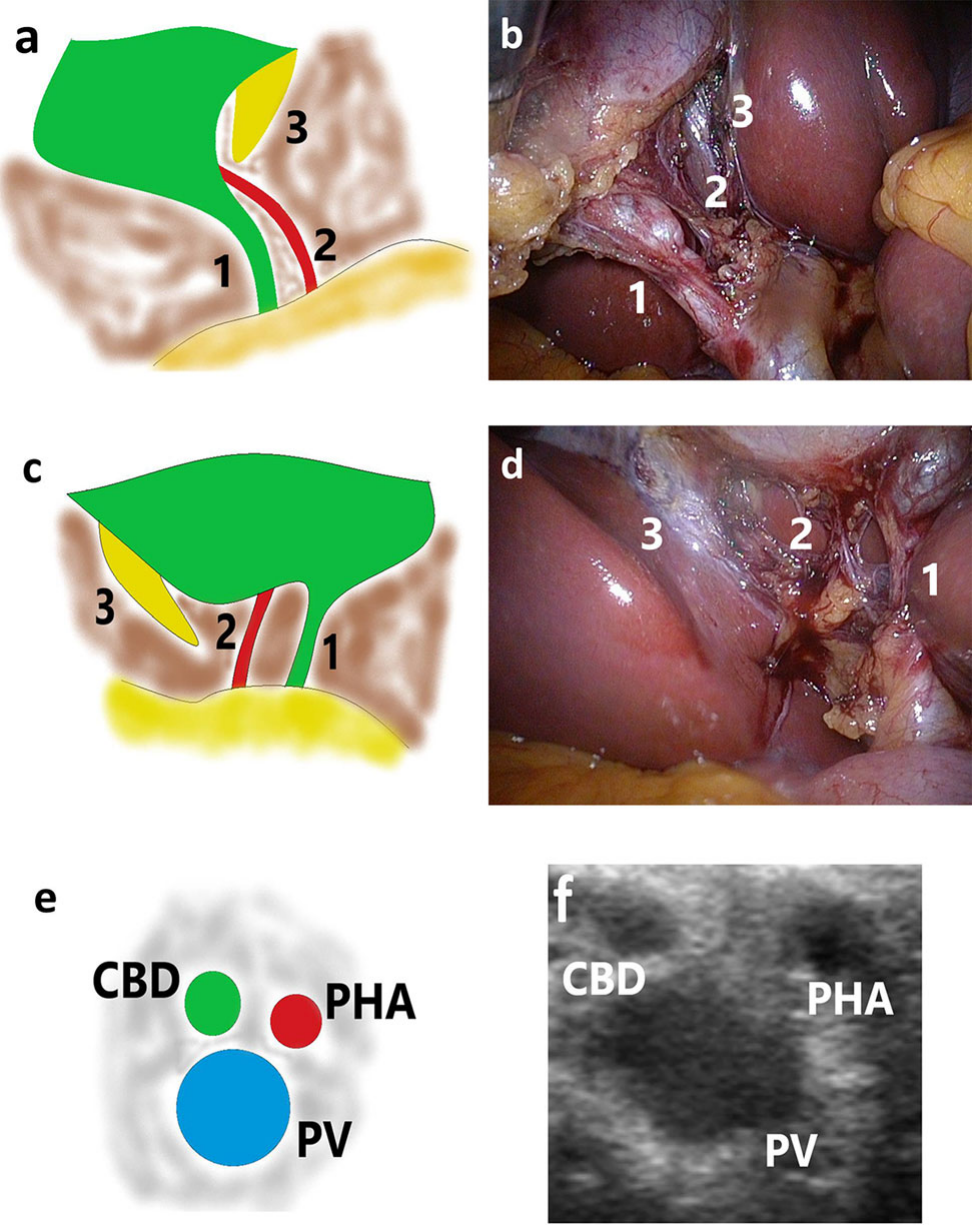
Photographic documentation of the critical view of safety and safe plane of dissection obtained with laparoscopic ultrasound. **a**, **b**-CVS anterior view, **c**, **d**-CVS posterior view, **e**, **f**-“Mickey-Mouse” sign. CBD-common bile duct, PHA-proper hepatic artery, PV-portal vein, CVS-critical view of safety, 1-cystic duct, 2-cystic artery, 3-cystic plate

**Fig. 2:**
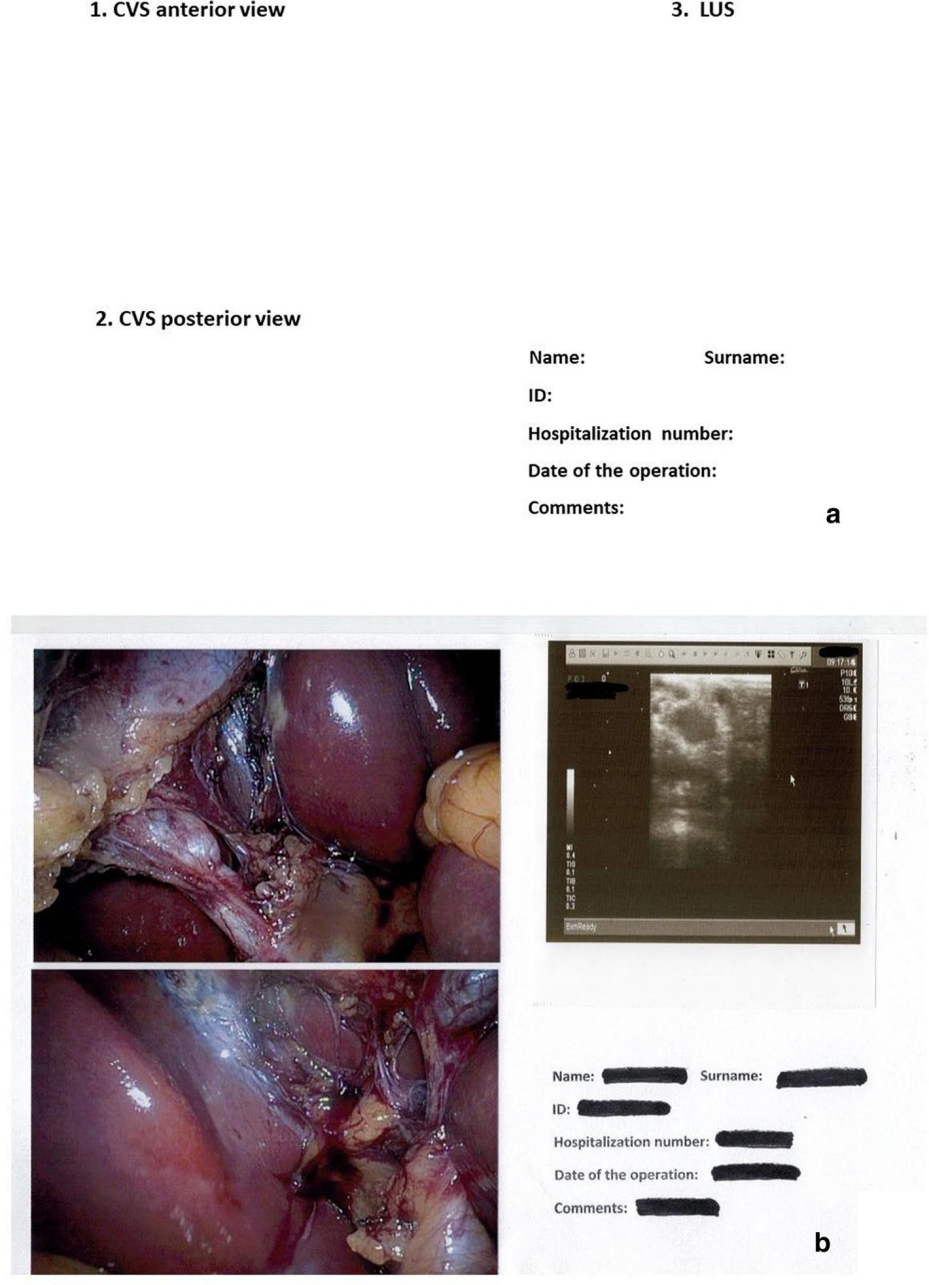
A4 sheet with the report of obtained CVS and “Mickey-Mouse” sign. **a**-blank sheet, **b**- ready sheet with photographs of CVS and safe plane of dissection. CVS-critical view of safety

Written informed consent was obtained from all patients before surgery. All procedures were in accordance with the ethical standards of the 1964 Declaration of Helsinki and its later amendments, and the study was reviewed and approved by the Ethical Committee of the Wroclaw Medical University (approval number BW-24/2020). Cholecystectomies were performed on an elective basis by two surgeons experienced in LC (> 150 cholecystectomies) and LUS (> 70 examinations). We used the laparoscopic probe Toshiba PEF-704 LA (frequency 7.0–10 MHz) and the diagnostic ultrasound system Toshiba NemioMX SSA-590A for LUS all manufactured in Japan. The CVS and LUS were performed routinely in every patient. Laparoscopic ultrasound probe was inserted through the epigastrical 10 mm (transverse view) or umbilical 10 mm trocar (longitudinal view). Vascular and avascular structures were differentiated with duplex doppler function. The key structure which was visualized throughout the procedure and defined the proper plane of dissection was the “Mickey Mouse sign”—a characteristic configuration of the bile duct, the proper hepatic artery and the portal vein in the hepatoduodenal ligament which is similar to the head of Mickey Mouse. The photographic documentation of both CVS from anterior (Fig. 1a, b) and posterior view (Fig. 1c and d) and LUS (Mickey Mouse sign) (Fig. 1e, f) was performed. When it was impossible to reach CVS, two photographs from laparoscopy and one of LUS before clipping and cutting key structures were taken. The photographs were printed on the Canon SELPHY CP1300 Wireless Compact Photo Printer (photograph size 148 × 100 mm), and LUS photograph was printed directly from ultrasound machine on printer Mitsubishi P93 (size 100 × 110 mm). The photographs were then stuck to the defined places on one A4 sheet with the data of the patient (Fig. 2a, b). The mean price of Canon printer is 120 American dollars ($), and the cost of one photograph is 0.25 cents and one ultrasound photograph costs 10 cents. The photographs were also stored as JPG files on hard disc of the computer. The video of the whole procedure was recorded on the external hard drive of the computer in format 720 × 480 MPG and in the study to compare it with photographs we included the length of the video till the CVS and safe plane of dissection were obtained. The price of external hard drive 1 terabyte (TB) (1 TB = 1000 gigabyte (GB), 1 GB = 1000 megabyte (MB)) to store the video is 50$ (5 cents for 1 GB). We counted the costs that the minimal usage time of the printer is 4 years (30$ per year, with 100 performed cholecystectomies/1 year it accounts for 30 cents/1 cholecystectomy) (source-www.amazon.com). We did not include the costs of ultrasound machine and ultrasound probe, ultrasound printer and computer because they are used for many other clinical purposes beyond this study. Statistical analysis included the Mann–Whitney U test for continuous variables. The level of statistical significance was set at 95% (*p *< 0.05).

## Results

CVS was successful in 91 patients and LUS in 99 patients. LUS enabled safe plane of dissection when all the three elements of CVS could not be reached, or there were indications for fundus-first or subtotal cholecystectomy. The conversion rate was 2%. The reason for conversion in both cases was indissectable fibrous tissue in the region of hepatoduodenal ligament, not the vascular injury, bleeding or BDI. One of these patients was converted in the early stage due to advanced chronic inflammation and impossibility to perform LUS and cholecystostomy was performed (Table 1). The mean time to obtain and analyse photographs was significantly shorter than video (5.68 vs. 26.69 min.), and the size of the stored data was significantly smaller for photographs than video (3.7 MB vs 3152 MB). The cost of one documentation procedure was significantly lower for video than photographs (0.16 vs. 0.90 $) (Table 2).

**Table 2:** Characteristics of photographs and video

Parameter	Time needed to obtain and analyse documentation [min]		Size of the stored data [MB]		Cost of one documentation procedure [$]	
	Photograph	Video	Photograph	Video	Photograph	Video
Mean	5.68	26.69	3.7	3152	0.90	0.16
Standard deviation	0.76	4.58	0.32	690.3		
Minimum	5	20	3.1	2301		
Maximum	7	42	4.4	4830		
Median	5.5	25	3.6	3210.5		
Significance	*P *< .0001^*^		*P *= 0.000000^*^		*P *= 0.000000^*^	

MB-megabyte

$-American dollar

*Statistically significant values (*P *< 0.05)

## Discussion

BDI still remains a dreaded complication following LC with plateaued rate of 0.08–0.3% [4, 5, 7, 15] and possible short-term morbidity of up to 40–50% and mortality of 2–4% [1, 5, 10, 16, 17]. Long-term complications may include anastomotic strictures, recurrent cholangitis, secondary biliary cirrhosis and impaired quality of life [5, 15]. Apart from the clinical outcomes, an important issue are also increased costs of hospitalization and medicolegal aspects [5, 8, 15, 18].

Vasculobiliary injury (VBI) is a concomitant biliary and vascular, especially arterial injury [19]. The right hepatic artery (RHA) injury is the most common VBI followed by injury to other arteries, the portal vein alone or in combination with arteries. It is concluded that isolated occlusion of the RHA not associated with biliary or portal vein injury rarely leads to clinically significant ischaemia to the bile ducts or liver [19]. Patients with a RHA VBI may develop a type of slow infarction of the right liver with the formation of zones that become infected leading to abscesses formation or ischaemic atrophy without reports of rapid complete infarction requiring emergency right hepatectomy. The portal vein injuries are rarely reported what may be associated with rapid liver infarction leading to patient deterioration and death before referral to a tertiary centre. The portal vein injuries may require an emergency right hepatectomy or liver transplant with 50% mortality rate in case of such injury. The similar sequel of events may be associated with injury to the proper hepatic artery or common hepatic artery. The authors concluded that future efforts in this area should be focused on prevention and one of the criteria for judging injury to a vascular structure may be intraoperative photographic or video documentation [19].

CVS is a part of the culture of safety in cholecystectomy (COSIC) and SAGES safe cholecystectomy program along with IOC and LUS to prevent BDI [8, 9]. More exposure and more dissection are needed to achieve all three elements of CVS with acute or chronic inflammation preventing its attainment what forces surgeon too chose among “bail-out” strategies such as “fundus-first” LC, subtotal LC or conversion [1]. Significant VBI may occur during “fundus-first” LC and a clear understanding of the anatomy of the cystic and hilar plates is required [20–23]. LUS is an ideal adjunct during dissection in dangerous area due to identification of safe plane of dissection with the visibility of vascular and avascular structures behind the inflammatory and fibrotic tissues. LUS may be repeated as many times as it is needed, and it is safe for both the patient and operating team [10]. The cannulation of the cystic duct and irradiation as in IOC or intravenous injection of indocyanine green as in near-infrared fluorescence cholangiography (NIRF-C) are not needed with at least the same success rate in visualization of bile duct with the advantage of recognition of vascular structures with the potential of protection against both BDI and VBI [10]. Mangieri et al. found that IOC use is still not protective against BDI, but it is difficult to make any definitive conclusions on this topic and cholecystitis is a risk factor of BDI. Conversion to an open procedure significantly increased the risk of BDI at almost 15% raising a question if it is still a safe “bailout” procedure what may be associated with the lack of experience among younger surgical generation and a technically difficult converted cases [4, 24]. Conversion rate of LC is 3–5%, but in a study of Mangieri et al., it was only 0.04% [4]. The proportion of open cholecystectomies in another study decreased from 2.6% in 2006 to 0.9% in 2011 [7]. In our study, we had 2% conversion rate with higher rate of “fundus-first” LCs what also reflects the trend towards laparoscopic “bailout” options than conversion.

Sanford and Strasberg proposed a simple and effective method to record the CVS during LC by intraoperative “doublet” photography. They stated that CVS should be considered the gold standard technique to assess biliary anatomy during LC, but there is a discrepancy in the rate of use and quality of CVS between surgeons what may be associated with the lack of visual record of attained CVS. Operatives notes of cases where BDI occurred and were treated by the authors were in fact not associated with achievement of CVS but with so-called “infundibular technique” resulting in “funnel view” being the error trap leading to BDI especially in the case of severe or acute inflammation [5, 8, 17, 25]. The authors also stated that videos are more expensive and difficult to store and that techniques of still photography of CVS should be optimized [16]. Buddingh et al. proposed correct photographic documentation of biliary anatomy with CVS and IOC. Both CVS and IOC were available for 63 patients. The inter-rater agreement for “conclusive” CVS was 27% and for “conclusive” IOC was 57% (*p *< 0.001). The history of cholecystitis diminished these values. The authors concluded that documentation of biliary anatomy is not as straightforward as it seems, the proportion of properly documented CVS is unacceptably low and that protocols are necessary especially for medicolegal purposes and laparoscopy training courses. The surgeon as well as the patient, public prosecutor and insurance company may use stored files in a trial, thus the photographic documentation should meet the appropriate standards because confirmation of obtaining CVS only in the operation notes may be in the future insufficient [13].

Another method of visual documentation of CVS are videos. Mascagni et al. proposed 60 s video segments prior to the division of cystic structures. CVS was attempted in 78 patients, but after evaluation and inter-rater agreement between two external surgeons, it was 32 out of 78 (41.03%) cases of LC. The authors concluded that it could be used for quality auditing, scientific communication and development of deep learning models for intraoperative guidance. They also stated that there should be a more automatic way to evaluate the safety check and an offer of intraoperative guidance [14]. Nijssen et al. investigated whether the criteria for CVS were met in complicated LCs. In the 65 analysed videos according to the operative notes, CVS was reached in 80% of cases, but in reality after evaluation by two investigators, it was only 10.8%, and CVS was not reached in any of the patients with BDI [26]. We found in our study that videos were significantly cheaper than photographs (which were both actually not expensive 0.90 cents/3 photographs vs 0.16 cents/1video), but time needed to analyse them was too long to perform it directly after the operation in the operating theatre what forced the surgeon to do it in any other possible time (surgeons usually lack of too much free time in their schedules). Another problem with videos and any other digital recording is that storage of data is not standardized and safe electronic copies are needed if they are going to be used in litigation process what doubles the costs of the procedure. In general, the significantly lower costs and potential ease of a video recording may be attractive for its supporters, but till the rules of their safe archivization and fast access to data are not explicitly determined, the clinical and legal values of videos are diminished. We did not see any discrepancies between the quality of CVS between photographs and videos because photographs were taken from the video of the same operation. The only advantage of the video seems to be the observation of the whole process which led to the obtaining CVS, but the end results were the same.

Alkhaffaf et al. analysed the trends in litigation following LC in England between 1995 and 2009. One hundred and ninety-eight (65%) out of 303 settled claims found to be in the claimants favour for a total cost of 20.4 million GPB (33.4 million USD). The only cause to result in a successful claim was the operator error (*P *= 0.023), and delay in the recognition of complications was the second most common reason for initiation of a claim. BDI was the most common injury leading to litigation and successful claim (*P *< 0.001) [27]. Perera et al. studied a group of 67 patients after major BDI following LC of whom 22 (33%) had started litigation. On multivariative analysis statistically significant (*P *< 0.05) predictors for possible litigation were age < 52 years, associated vascular injury, immediate nonspecialist repair and perceived incomplete recovery following BDI. On the other hand, factors which prevented patients to pursue legal action were the lack of trust in the health care system, legal opinion suggesting lack of strong evidence, injury associated mainly with human error and satisfaction with injury management [28].

The limitation of our study was a relatively small study group, the data only from one Department of Surgery and only one type of equipment used to perform LUS and print the photographs, thus further studies including larger groups of patients in more surgical centres and comparison of available equipment are needed in order to strengthen our findings, especially in protection against BDI and VBI and usefulness during the litigation process.

To conclude, proper and high-quality documentation of both CVS and LUS should be an inherent part of cholecystectomy. Our proposal of documentation consists of two photographs of CVS and one photograph of LUS printed in the operating theatre and stored on one A4 sheet in the patient’s documentation. It is an easy, fast and cheap method along with carefully documented and detailed operative notes on every case, which may be use not only for data archivization but also for teaching, especially young surgeons, and medicolegal purposes as a strong evidence of obtaining CVS and proper plane of dissection with the potential of protection against both BDI and VBI. As with any operation, complications including BDI and VBI will occur during cholecystectomy, and it is the responsibility of the surgeon to do as much as he or she is able to ensure patient safety.
